# The goose of the golden eggs and the anatomy of a crisis: inflation, avian influenza, and the pandemic specter

**DOI:** 10.1590/0102-311XEN097625

**Published:** 2025-11-07

**Authors:** Ivi Vasconcelos Elias

**Affiliations:** 1 Escola de Comando e Estado Maior do Exército, Rio de Janeiro, Brasil.

The metaphor of the “goose of the golden eggs” expresses how the overexploitation of resources can lead to ruin. Recent avian flu outbreaks concretize this image by articulating three phenomena: egg price inflation in Brazil, the recurrence of the disease, and the risk of new pandemics. This article examines the intersections between food security, global health, and agribusiness, in light of the political economy and poultry production chains in the United States and Brazil. It argues that the intensification of avian flu, combined with the fragility of biosecurity and the volatility of global markets, transforms industrial poultry farming into a critical point of systemic crises. The text is organized around three axes: the social nature of the avian flu virus, the dynamics of the international egg trade, and the impacts on Brazil and the United States. In the first case, high productivity and concentration increase vulnerability to outbreaks and zoonotic spillover. In the second, a booming foreign market strengthens Brazilian production but puts pressure on domestic prices, exacerbating inflation and food insecurity. Two central paradoxes emerge from this analysis: the more efficient and concentrated the production, the greater the vulnerability to health shocks; and the more booming the foreign market, the greater the negative impact on domestic consumption. Thus, the productive logic of agribusiness converts immediate gains into structural risks for society.

## Avian influenza: the social side of the virus

Influenza A viruses pose a serious threat to public health due to the high mutation rate and potential to become a pandemic. Historically, these viruses have been responsible for pandemics such as the Spanish flu (1918, H1N1), the Asian flu (1957, H2N2), the Hong Kong flu (1968, H3N2), and the swine flu (2009, H1N1) ^1^. In 2020, a new H5N1 variant became predominant in Africa, Europe, and the Middle East. It was detected in North America in late 2021, with outbreaks on commercial poultry farms in the United States beginning in February 2022 ^2^. Between 2003 and January 2025, the World Health Organization (WHO) reported 964 human cases of H5N1 in 24 countries, resulting in 466 deaths. More cases appeared in the Americas starting in 2022, with 71 infections reported by February 2025 - 70 in the United States and 1 in Canada. In the same period, the virus is estimated to have caused the death of approximately 130 million birds in the United States. In South America, the deaths of approximately 600,000 wild birds and 50,000 mammals have been recorded ^3^.

Given this transcontinental spread, avian influenza began to be characterized as a panzootic - an animal epidemic that affects multiple species in different regions of the world. The occurrence of infections in mammals has attracted increasing attention, as it may signal a possible adaptation of the virus to humans. The interface between wildlife, domesticated animals, and humans, especially in intensive agro-industrial systems, constitutes an environment conducive to cross-transmission and the leap between species. Factors such as high population density, confinement, and the circulation of workers increase the risk of viral dissemination. It is not by chance that most human cases of H5N1 have occurred in occupational settings involving direct contact with infected animals.

Although attention is often focused on areas of outbreak, it is essential to consider the social, economic, and agroecological relationships that connect economic actors on a global scale ^4^. The cause of new pandemics is not found only in the clinical course of viruses, but in the convergence between pathogenic mechanisms and sociocultural processes. This interconnection is at the core of the emergence of contemporary pandemics.

Viruses must also be understood as social facts, the spread of which results from the interaction between biology and the historical and economic contexts of agricultural production. Far from being natural phenomena, outbreaks express socially determined causalities rooted in the logic of capital. Agribusiness transforms nature into a mechanism for producing value, even subjecting pathogens to the dynamics of accumulation. Thus, viruses are part of the political economy of food in networks that connect human actions, globalized chains, and ecological processes. Under these conditions, production chains themselves become vectors of disease, increasing the risk of zoonotic spillover.

## Poultry production chains and avian influenza

The international egg trade has historically been characterized by a low volume of transactions due to the perishable nature of the product and the preference for local supply chains. The main global producers also account for the largest consumption, which limits the need for large-scale importation and exportation ^5^. This pattern, however, began to change with the effects of avian influenza, which destabilized the supply and increased the demand for external suppliers.

The United States, which is one of the epicenters of the current health crisis and a major egg producer on the global stage, has a poultry market characterized by high concentration and vertical integration, with companies controlling all steps of the chain - from feed production to distribution. The five largest companies account for approximately 46% of commercial hens, with Cal-Maine Foods corresponding to approximately 20% of the US production ^5^. At the same time, the average scale of production units has grown and farms with more than one million birds are commonplace. Although this model has led to substantial gains in productivity, it has also intensified challenges related to animal welfare, environmental sustainability, and biosafety.

The structural characteristics of the poultry industry in the United States - marked by high concentration, vertical integration, and dependence on large farms - make the sector particularly vulnerable to external shocks, such as outbreaks of H5N1. This situation has resulted in a drop in the egg supply, price inflation, and an increase in imports. The U.S. Department of Agriculture (USDA) projected an increase of up to 41% in egg prices by 2025 ^6^. In response, the USDA announced a USD 1 billion investment plan aimed at containing avian influenza and stabilizing the market. Of this amount, USD 500 million will be allocated to biosafety measures, USD 400 million to direct financial support for producers, and USD 100 million to vaccine research ^6^. This demonstrates that the government is called upon to intervene in health emergencies, even in strongly neoliberal contexts.

The avian influenza has also affected other major producers, such as the European Union and Japan, which responded to the crisis by expanding their importation possibilities. This international situation drove the demand for Brazilian products and boosted the national export sector. In April 2025, Brazil exported 4,300 tons of eggs, which is a 271% increase compared to the volume recorded in the same month of 2024. Exports to the European Union grew by 64% in the same comparison and sales to Japan increased by 111.3%. The United States led the international demand for Brazilian eggs, with an impressive 816% increase in the volume exported between January and April 2025 compared to the same period the previous year ^7^. Between 2015 and 2023, Brazil stood out as one of the countries with the highest growth in egg production, increasing its participation in the international market and assuming the fifth position among the world’s largest producers ^8^.

The insertion of the Brazilian egg market into international trade has increased the concentration of production, with the expansion of industrial poultry farming and the contraction of medium-sized production ^9^. This movement brings the sector closer to the trend seen in the United States, where the already consolidated concentration increases efficiency but also increases vulnerability to external shocks. At the same time, production for internal consumption is also growing, with approximately 99% of production destined for the domestic market ^10^, highlighting the importance of eggs to the food security of the population. Paradoxically, the increase in production directed towards the foreign market strengthens the sector but puts pressure on domestic prices. The price of eggs rose 13.13% in March 2025 and is one of the main drivers of the 0.56% monthly inflation rate, which, in turn, impacts food security by restricting the population’s access to this food product ^11^.

Since the initial detection of the virus in Brazil in May 2023, 185 outbreaks have been identified, 172 of which were in wild birds, 12 were on subsistence farms, and one was on a commercial farm ^12^. As a preventive measure, the Brazilian Ministry of Agriculture and Livestock (MAPA, acronym in Portuguese) ^13^ declared a state of animal health emergency through *Ordinance n. 782/2025*, which was extended for another 180 days by *Ordinance n. 784/2025.* As a strategic response, the Butantan Institute developed the first Brazilian avian flu vaccine for human use, which is still in the clinical testing phase ^14^. Unlike what occurred during the COVID-19 pandemic, the aim of this measure is to ensure technological autonomy and national response capacity in the face of a potential pandemic emergency. Even with an effective vaccine, however, challenges remain, such as large-scale production, distribution logistics, ensuring equitable access to immunization, and coordinating public health policies to contain the spread of the virus.

On the international level, the WHO, World Organization for Animal Health (WOAH), and Food and Agriculture Organization of the United Nations (FAO) play a central role in coordinating surveillance, containment, and genetic data-sharing on avian influenza in accordance with the International Health Regulations (IHR). The FAO and WHO launched a new global strategy for the prevention and control of the highly pathogenic avian influenza based on the “One Health” approach, with the aim of strengthening the resilience of agri-food systems and mitigating impacts on animal production and trade chains ^15^. The signing of the WHO Agreement on Pandemic Prevention, Preparedness, and Response - the text of which was finalized in April 2025 - is also expected ^16^. However, the withdrawal of the United States from the organization at the start of the new Trump administration in 2025 constitutes a significant obstacle to international coordination for the management of future pandemics.

## Conclusion

In summary, the recent outbreaks of avian influenza and the economic and health impacts demonstrate the interdependence of agrifood production models, food security, and public health risks. The intensification of production directed towards the foreign market increases vulnerability to pathogens and compromises the domestic supply, exacerbating inequalities in access to food. The increase in the price of eggs in Brazil amid expanding exports reveals this paradox. Although the “One Health” approach represents progress, it has operated reactively, failing to address the structural factors associated with intensive agro-industrial systems that favor the occurrence of health emergencies. The metaphor of the goose that lays the golden eggs becomes concrete: by prioritizing productivity and immediate profit, the system threatens the very foundation that sustains it. Overcoming this impasse requires more than the control of outbreaks; it requires the transformation of the modes of food production and distribution, with a focus on social justice, food sovereignty, and collective health.

## References

[1] Wille M, Barr IG (2022). Resurgence of avian influenza virus. Science.

[2] Centers for Disease Control and Prevention Emergence and evolution of H5N1 bird flu..

[3] Organização Pan-Americana da Saúde Atualização epidemiológica: influenza aviária A (H5N1) na Região das Américas, 4 de março de 2025..

[4] Wallace R (2020). Pandemia e agronegócio: doenças infecciosas, capitalismo e ciência.

[5] Amaral GF, Guimarães DD, Nascimento JCOF, Custodio S (2016). Avicultura de postura estrutura da cadeia produtiva, panorama do setor no Brasil e no mundo e o apoio do BNDES. BNDES Setorial.

[6] U.S. Department of Agriculture USDA invests up to $1 billion to combat avian flu and reduce egg prices..

[7] Associação Brasileira de Proteína Animal Exportações de ovos crescem em abril e Brasil amplia presença em novos mercados..

[8] Food and Agriculture Organization of the United Nations FAOSTAT: crops and livestock products..

[9] Henn JD, Miele M, Almeida MMTB Caracterização da avicultura comercial de pequena escala e a regularização das granjas. Comunicado Técnico nº 606..

[10] Associação Brasileira de Proteína Animal Relatório anual 2025..

[11] Instituto Brasileiro de Geografia e Estatística (2025). Inflação fica em 0,56% em março, pressionada por alimentos.. Agência IBGE Notícias.

[12] Ministério da Agricultura e Pecuária https://mapa-indicadores.agricultura.gov.br/publico/extensions/SRN/SRN.html.

[13] Ministério da Agricultura e Pecuária Ministério da Agricultura e Pecuária confirma primeiro foco de gripe aviária em granja comercial no Brasil..

[14] Fiocruz Pernambuco (2025). Fiocruz e Butantan farão estudo clínico da primeira vacina brasileira contra gripe aviária.. Agência Fiocruz de Notícias.

[15] Food and Agriculture Organization of the United Nations; World Organisation for Animal Health Global strategy for the prevention and control of high pathogenicity avian influenza (2024-2033)..

[16] Organização Mundial da Saúde Estados Membros da OMS concluem negociações e fazem progressos significativos na proposta de acordo sobre pandemia..

