# Supplementary material for: The goose of the golden eggs and the anatomy of a crisis: inflation, avian influenza, and the pandemic specter
Source: Cad Saude Publica. 2025 Nov 7;41(10):e00097625. doi: 10.1590/0102-311XEN097625 (PMC12600014; doi:10.1590/0102-311XEN097625)
Supplement: Translation

# Translation: A galinha dos ovos de ouro e a anatomia de uma crise: inflação, gripe aviária e o espectro pandêmico

**DOI:** 10.1590/0102-311XPT097625

**Authors:** Ivi Vasconcelos Elias

**Affiliations:** 1 Escola de Comando e Estado Maior do Exército, Rio de Janeiro, Brasil.

A metáfora da “galinha dos ovos de ouro” expressa como a exploração excessiva de recursos pode levar à própria ruína. Os surtos recentes de gripe aviária concretizam essa imagem ao articular três fenômenos: a inflação do preço dos ovos no Brasil, a recorrência da doença e o risco de novas pandemias. Este artigo examina as interseções entre segurança alimentar, saúde global e agronegócio, à luz da economia política e das cadeias de produção avícolas de Estados Unidos e Brasil. Sustenta-se que a intensificação da gripe aviária, aliada à fragilidade da biossegurança e à volatilidade dos mercados globais, transforma a avicultura industrial em um ponto crítico das crises sistêmicas. O texto organiza-se em três eixos: o caráter social do vírus da gripe aviária, a dinâmica do comércio internacional de ovos e os impactos sobre Brasil e Estados Unidos. No primeiro caso, a alta produtividade e concentração ampliam a vulnerabilidade a surtos e ao transbordamento zoonótico; no segundo, o aquecimento do mercado externo fortalece a produção brasileira, mas pressiona os preços internos, agravando a inflação e a insegurança alimentar. Dessa análise emergem dois paradoxos centrais: quanto mais eficiente e concentrada a produção, maior a vulnerabilidade a choques sanitários; e quanto mais aquecido o mercado externo, maior o impacto negativo sobre o consumo doméstico. Assim, a lógica produtiva do agronegócio converte ganhos imediatos em riscos estruturais para a sociedade.

## Gripe aviária: o lado social do vírus

Os vírus da influenza A representam séria ameaça à saúde pública devido à alta taxa de mutação e ao potencial pandêmico. Historicamente, foram responsáveis por pandemias como a gripe espanhola (1918, H1N1), a asiática (1957, H2N2), a de Hong Kong (1968, H3N2) e a gripe suína (2009, H1N1) ^1^. Em 2020, uma nova variante do H5N1 tornou-se predominante na África, Europa e Oriente Médio sendo detectada na América do Norte no final de 2021, com surtos em granjas comerciais dos Estados Unidos a partir de fevereiro de 2022 ^2^. Entre 2003 e janeiro de 2025, a Organização Mundial da Saúde (OMS) registrou 964 casos humanos de H5N1 em 24 países, com 466 mortes. A partir de 2022, os casos aumentaram nas Américas, com 71 infecções relatadas até fevereiro de 2025, sendo 70 nos Estados Unidos e 1 no Canadá. No mesmo período, estima-se que o vírus tenha causado a morte de cerca de 130 milhões de aves nos Estados Unidos. Na América do Sul, registraram-se aproximadamente 600 mil mortes de aves silvestres e 50 mil de mamíferos ^3^.

Diante dessa disseminação transcontinental, a gripe aviária passou a ser caracterizada como uma panzootia, isto é, uma epidemia animal que afeta múltiplas espécies em diferentes regiões do mundo. A ocorrência de infecções em mamíferos tem sido objeto de atenção crescente, pois pode sinalizar uma possível adaptação do vírus aos humanos. A interface entre fauna silvestre, animais de criação e seres humanos, especialmente em sistemas agroindustriais intensivos, constitui um ambiente propício à transmissão cruzada e ao salto interespecífico. Fatores como alta densidade populacional, confinamento e circulação de trabalhadores aumentam os riscos de disseminação viral. Não por acaso, a maioria dos casos humanos de H5N1 ocorreu em contextos ocupacionais envolvendo contato direto com animais infectados.

Embora a atenção frequentemente se concentre nas zonas dos surtos, é imprescindível considerar as relações sociais, econômicas e agroecológicas que conectam atores econômicos em escala global ^4^. Assim, a causa das novas pandemias não se encontra apenas no curso clínico dos vírus, mas na convergência entre mecanismos patogênicos e processos socioculturais, sendo essa articulação o cerne do surgimento das pandemias contemporâneas.

Os vírus devem ser entendidos também como fatos sociais, cuja disseminação resulta da interação entre biologia e contextos históricos e econômicos da produção agrícola. Longe de fenômenos naturais, os surtos expressam causalidades socialmente determinadas, enraizadas na lógica do capital. O agronegócio transforma a natureza em mecanismo de produção de valor, submetendo inclusive patógenos à dinâmica da acumulação. Assim, os vírus integram a economia política dos alimentos, em redes que articulam ações humanas, cadeias globalizadas e processos ecológicos. Nessas condições, as próprias cadeias produtivas tornam-se vetores de doenças, ampliando o risco de transbordamento zoonótico.

## Cadeias produtivas avícolas e a gripe aviária

O comércio internacional de ovos tem sido historicamente caracterizado por um volume reduzido de transações, devido à perecibilidade do produto e à preferência por cadeias de abastecimento locais. Os principais produtores globais concentram também o maior consumo, o que limita a necessidade de importações e exportações em larga escala ^5^. Esse padrão, no entanto, começou a se modificar com os efeitos da gripe aviária, que desestabilizaram a oferta e ampliaram a demanda por fornecedores externos.

Os Estados Unidos, um dos epicentros da atual crise sanitária e grande produtor mundial de ovos, possui um mercado avícola marcado por alta concentração e integração vertical, com empresas controlando todas as etapas da cadeia, da produção de ração à distribuição. As cinco maiores companhias respondem por cerca de 46% das galinhas poedeiras comerciais, sendo a Cal-Maine Foods responsável por aproximadamente 20% da produção estadunidense ^5^. Paralelamente à concentração, observa-se o crescimento da escala média das unidades produtivas, sendo comum a existência de granjas com mais de um milhão de aves. Embora esse modelo tenha promovido ganhos expressivos em produtividade, também intensificou desafios relacionados ao bem-estar animal, à sustentabilidade ambiental e à biossegurança.

As características estruturais da avicultura nos Estados Unidos, marcada por alta concentração produtiva, integração vertical e dependência de grandes granjas, tornam o setor especialmente vulnerável a choques externos como os surtos de H5N1. Essa conjuntura resultou em queda na oferta de ovos, inflação nos preços e aumento das importações. O Departamento de Agricultura dos Estados Unidos (USDA, acrônimo em inglês) projeta um aumento de até 41% nos preços dos ovos em 2025 ^6^. Como resposta, o USDA anunciou um plano de investimento de USD 1 bilhão, com o objetivo de conter a gripe aviária e estabilizar o mercado. Desse montante, USD 500 milhões serão destinados a medidas de biossegurança, USD 400 milhões ao apoio financeiro direto a produtores e USD 100 milhões à pesquisa de vacinas ^6^, evidenciando que o Estado é chamado a intervir frente a emergências sanitárias, mesmo em contextos fortemente neoliberais.

Os efeitos da gripe aviária atingiram também outros grandes produtores, como a União Europeia e o Japão, que responderam à crise ampliando suas possibilidades de importação. Essa conjuntura internacional impulsionou a demanda por produtos brasileiros e aqueceu o setor exportador nacional. Em abril de 2025, o Brasil exportou 4,3 mil toneladas de ovos, volume 271% superior ao registrado no mesmo mês de 2024. As exportações destinadas à União Europeia apresentaram crescimento de 64% no mesmo comparativo, enquanto as vendas ao Japão aumentaram 111,3%. Os Estados Unidos lideraram a demanda internacional por ovos brasileiros, com uma elevação expressiva de 816% no volume exportado entre janeiro e abril de 2025, em relação ao mesmo período do ano anterior ^7^. Entre 2015 e 2023, o Brasil destacou-se como um dos países com maior crescimento na produção de ovos, aumentando sua participação no mercado internacional e assumindo a quinta posição entre os maiores produtores mundiais ^8^.

A inserção do mercado brasileiro de ovos no comércio internacional tem ampliado a concentração produtiva, com expansão da avicultura industrial e retração da produção de médio porte ^9^. Esse movimento aproxima o setor da tendência observada nos Estados Unidos, onde a concentração já consolidada aumenta a eficiência, mas também a vulnerabilidade a choques externos. Paralelamente, cresce a produção voltada ao autoconsumo, sendo que cerca de 99% da produção permanece destinada ao mercado interno ^10^, evidenciando a importância dos ovos para a segurança alimentar da população. Em um paradoxo, o aumento da produção orientada ao mercado externo fortalece o setor, mas pressiona os preços internos. Em março de 2025, os ovos de galinha subiram 13,13%, sendo um dos principais vetores da inflação mensal de 0,56%, o que, por sua vez, impacta a segurança alimentar restringindo o acesso da população a esse alimento ^11^.

No Brasil, desde a detecção inicial do vírus, em maio de 2023, foram identificados 185 focos, dos quais 172 em aves silvestres, 12 em criações de subsistência e 1 em criação comercial ^12^. Como medida preventiva, o Ministério da Agricultura e Pecuária (MAPA) ^13^ declarou estado de emergência zoossanitária por meio da *Portaria nº 782/2025*, prorrogada por mais 180 dias pela *Portaria nº 784/2025*. Em resposta estratégica, o Instituto Butantan desenvolveu a primeira vacina brasileira contra a gripe aviária para uso humano ainda em fase de testes clínicos ^14^. Ao contrário do ocorrido durante a pandemia de COVID-19, a medida visa assegurar autonomia tecnológica e capacidade nacional de resposta diante de uma possível emergência pandêmica. No entanto, mesmo com uma vacina eficaz, persistem desafios como a produção em larga escala, a logística de distribuição, a garantia de acesso equitativo à imunização e a articulação de políticas públicas de saúde para conter a propagação do vírus.

No plano internacional, a OMS, a Organização Mundial da Saúde Animal (OMSA) e a Organização das Nações Unidas para Alimentação e Agricultura (FAO, acrônimo em inglês) exercem papel central na coordenação das ações de vigilância, contenção e compartilhamento de dados genéticos sobre a influenza aviária, em conformidade com o Regulamento Sanitário Internacional (RSI). Nesse contexto, FAO e OMSA lançaram uma nova estratégia global para a prevenção e o controle da influenza aviária altamente patogênica, fundamentada na abordagem “Uma Só Saúde”, com o objetivo de fortalecer a resiliência dos sistemas agroalimentares e mitigar impactos sobre as cadeias produtivas e comerciais da produção animal ^15^. Há ainda a expectativa de assinatura do Acordo sobre Prevenção, Preparação e Resposta a Pandemias da OMS, cujo texto foi concluído em abril de 2025 ^16^. A retirada dos Estados Unidos da organização no início do novo governo Trump em 2025, porém, representa um obstáculo significativo à articulação internacional para a gestão de futuras pandemias.

## Conclusão

Em síntese, os surtos recentes de gripe aviária e seus impactos econômicos e sanitários evidenciam a interdependência entre os modelos de produção agroalimentar, a segurança alimentar e os riscos à saúde pública. A intensificação produtiva voltada ao mercado externo amplia a vulnerabilidade a patógenos e compromete o abastecimento interno, agravando desigualdades no acesso aos alimentos. O aumento dos preços dos ovos no Brasil, em meio à expansão das exportações, revela esse paradoxo. Embora a abordagem “Uma Só Saúde” represente um avanço, tem operado de forma reativa, sem enfrentar os fatores estruturais associados aos sistemas agroindustriais intensivos que favorecem o surgimento de emergências sanitárias. A metáfora da galinha dos ovos de ouro torna-se concreta: ao priorizar a produtividade e o lucro imediato, o sistema ameaça a própria base que o sustenta. Superar esse impasse exige mais que controle de surtos; requer a transformação dos modos de produção e distribuição de alimentos, com foco em justiça social, soberania alimentar e saúde coletiva.
